# Low**-**Dimensional Halide Perovskites and Their Advanced Optoelectronic Applications

**DOI:** 10.1007/s40820-017-0137-5

**Published:** 2017-03-13

**Authors:** Jian Zhang, Xiaokun Yang, Hui Deng, Keke Qiao, Umar Farooq, Muhammad Ishaq, Fei Yi, Huan Liu, Jiang Tang, Haisheng Song

**Affiliations:** 1 Wuhan National Laboratory for Optoelectronics (WNLO), Huazhong University of Science and Technology (HUST), 1037 Luoyu Road, Wuhan, 430074 Hubei People’s Republic of China; 2 School of Optical and Electronic Information, Huazhong University of Science and Technology (HUST), 1037 Luoyu Road, Wuhan, 430074 Hubei People’s Republic of China

**Keywords:** Metal halide perovskites, Low-dimensional effect, Synthesis, Optoelectronic devices, Versatility

## Abstract

Metal halide perovskites are crystalline materials originally developed out of scientific curiosity. They have shown great potential as active materials in optoelectronic applications. In the last 6 years, their certified photovoltaic efficiencies have reached 22.1%. Compared to bulk halide perovskites, low-dimensional ones exhibited novel physical properties. The photoluminescence quantum yields of perovskite quantum dots are close to 100%. The external quantum efficiencies and current efficiencies of perovskite quantum dot light-emitting diodes have reached 8% and 43 cd A^−1^, respectively, and their nanowire lasers show ultralow-threshold room-temperature lasing with emission tunability and ease of synthesis. Perovskite nanowire photodetectors reached a responsivity of 10 A W^−1^ and a specific normalized detectivity of the order of 10^12^ Jones. Different from most reported reviews focusing on photovoltaic applications, we summarize the rapid progress in the study of low-dimensional perovskite materials, as well as their promising applications in optoelectronic devices. In particular, we review the wide tunability of fabrication methods and the state-of-the-art research outputs of low-dimensional perovskite optoelectronic devices. Finally, the anticipated challenges and potential for this exciting research are proposed.

## Introduction

Low-dimensional materials are nanocrystals with at least one dimension within the nanoscale range (1–100 nm). For the last few decades, they have been increasingly attracting interest as they exhibit unique properties. They are also referred to as artificial atoms because the density of their electronic states can be widely and easily tuned by adjusting the crystal composition, size, shape, and so on. A typical example of the effect of size on crystal properties is quantum dots (QDs), whose three dimensions are all in the nanoscale range. The bandgaps could be easily tuned by adjusting the dot size [1]. A variety of optoelectronic applications have been explored for low-dimensional perovskites such as photovoltaics, light sensors, light-emitting diodes (LEDs), owing to the strong light–matter interaction [2]. Unlike traditional semiconductor nanomaterials, low-dimensional halide perovskites can be prepared at low cost and by solution-processable techniques. They have demonstrated superior optical, magnetic, dielectric, electrical, and optoelectronic properties [3–5] and have been becoming a hot research topic in new semiconductor materials and optoelectronic devices.

**Fig. 1:**
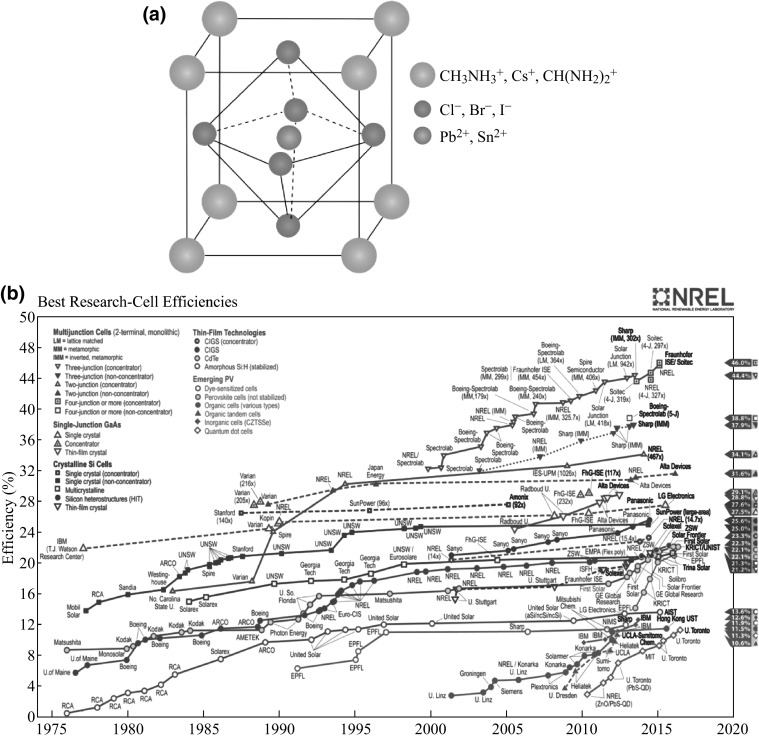
Perovskite structure and solar cell efficiencies. **a** Perovskites possess the general crystal structure ABX_3_. The most prevalent perovskite in optoelectronic devices is MA lead trihalide, for which *A* = CH_3_NH_3_, *B* = Pb, and *X* = Cl, Br or I. **b** Best research cell efficiencies. Adapted image reproduced from NREL [6]

The origin of “Perovskite” is calcium titanate (CaTiO_3_), which was discovered by Russian mineralogist L.A. Perovski in 1839. After that, perovskite was defined as a class of compounds that have the same crystal structure as CaTiO_3_, known as the perovskite structure [7]. The general chemical formula for pure perovskite compounds is ABX_3_ (Fig. 1a) where “A” and “B” are two cations of dissimilar size, and X is an anion that binds to both. Chalcogenide perovskites (AMO_3_) are formed from divalent A^II^ (Mg^2+^, Ca^2+^, Sr^2+^, Ba^2+^, Pb^2+^) and tetravalent M^IV^ (Ti^4+^, Si^4+^, Fe^4+^) elements with O^2−^ as the chalcogenide anion. Halide perovskite (AMX_3_) represents a large collateral series of the perovskite family, and it is reasonable to divide them roughly into alkali–halide perovskite and organometal halide perovskite. The first category is mainly formed from the monovalent cations (Li^+^, Na^+^, K^+^, Rb^+^, Cs^+^; aliphatic or aromatic ammonium) and the divalent M^II^ (Be^2+^, Mg^2+^, Ca^2+^, Sr^2+^, Ba^2+^, Zn^2+^, Ge^2+^, Sn^2+^, Pb^2+^, Fe^2+^, Co^2+^, Ni^2+^) with X representing halogen anions (F^−^, Cl^−^, Br^−^, I^−^). Oxide-based perovskites have been extensively studied, owing to their superior ferroelectric, magnetic, and superconductive properties [8]. The first halide-based perovskite structure was observed in cesium lead halides (CsPbX_3_) by Moller in 1958. Their photoconductive properties could be tuned through varying halide components to achieve different spectral responses. The first appearance of the organic cation, methylammonium (MA), in halide perovskites was studied by Weber and Naturforsch in 1978 [9, 10].

In the 1990s, Mitzi [11] focused on layered organic–inorganic halide perovskites that featured strong excitonic characteristics and demonstrated applications in thin-film transistors and LEDs. After Miyasaka et al.’s pioneered work in sensitized solar cells, the hybrid perovskite began its debut in photovoltaics in 2006 [12]. In 2009, a power conversion efficiency (PCE) of 3.8% was achieved by replacing Br with I [13]. In 2011, Park and coworkers achieved an efficiency of 6.5% by employing perovskite QDs as the sensitizers [14]. Later, Snaith et al. reported an efficiency of 10.9% utilizing solid electrolyte as a hole transport layer (HTL) [15]. As the study progressed, the superior properties of perovskites unfolded. Ambipolar carrier transport enlightened and promised the intensive employment of planar heterojunction devices [16–18]. This sparked an enormous development in the hybrid lead perovskite solar cells, which obtained a record PCE up to 22.1% in just 6 years using a low-cost solution method (Fig. 1b). In addition to PV applications, low-dimensional perovskite crystals with specific morphologies and ultrasmall size evoked new research interest in optoelectronic applications.

Compared with the hot research into halide perovskite thin-film solar cells, the low-dimensional perovskites hold great potential and just open a small angle. Thus, the present review mainly focuses on low-dimensional halide perovskite to summarize their synthesis methods, optoelectronic applications, and development outlook. By analogy to the human lifetime, the present stage of research into low-dimensional halide perovskites is just in its infancy. The high quantum yield, narrow full-width-at-half-maximum (FWHM), and tunable emission color of low-dimensional perovskite materials make them bright prospects for novel optoelectronic devices. A variety of monochromatic LEDs have been fabricated at room temperature utilizing inorganic cesium lead halide perovskite QDs as the color conversion layer, which have exhibited the best perovskite LED performance so far. Simultaneously, a hybrid perovskite nanowire photodetector (PD) with a photoconductive gain approaching 10^14^ electrons per photon and a responsivity approaching 6.0 × 10^5^ A W^−1^ has been developed. Single-crystal lead halide perovskite nanowire lasers exhibited ultralow lasing thresholds (220 nJ cm^−2^) and high quality factors (Q ∼ 3600) at a charge carrier density of ~1.5 × 10^16^ cm^−3^. As hybrid perovskite researchers investigate more deeply, many amazing properties are expected to be uncovered beyond photovoltaics.

In this review, we present a thorough treatment of the recent developments in the fundamental material properties of low-dimensional perovskites (QDs, nanowires, and nanosheets). We mainly focus on the synthesis, unique properties, and notable breakthroughs of perovskites, with optoelectronic applications in photovoltaics, LEDs, PDs, and lasers. Finally, the near-future challenges and the potential directions of this exciting research area are forecasted.

## Crystal Structure and Tunability of Halide Perovskite

Perovskites, with general formula AMX_3_, are a well-known class of inorganic materials with widespread deployment in ferro- and piezoelectric, magnetoresistive, semiconducting, and catalysis applications. The rich diversity is attainable owing to the multitude of large bivalent cations occupying the A-site (e.g., Ca^2+^, Sr^2+^, Ba^2+^), the smaller tetravalent cations (e.g., Ti^4+^, Zr^4+^) at the M site, and oxygen anions located at the X-site. Halide perovskites are different from their classical chalcogenide counterparts with monovalent moieties introduced at the A-site, divalent metal cations at the M site, and typically halides at the X-site. The AMX_3_ hybrid perovskite structure is formed by a three-dimensional (3D) network, with A-site cations occupying the cavity between four adjacent corner-sharing MX_6_ metal halide octahedra (e.g., *M* = Pb^2+^, Sn^2+^, Ge^2+^, Cu^2+^, Eu^2+^, Co^2+^ and *X* = Cl^−^, Br^−^, I^−^). The probability of forming the perovskite structure can be estimated using the Goldschmidt tolerance factor (*t*) and the octahedral factor (*µ*) [12, 19, 20]. Here, *t* is based on the ionic radii (*r*) of the A, M, and X, constituenting in $t = \left( {r_{A} + r_{X} } \right)/\sqrt 2 \cdot \left( {r_{M} + r_{X} } \right)$, and *µ* is defined as *r*_M_/*r*_*X*_. According to the tolerance factor, only the incorporation of small cations results in perovskite formation (*t* ~ 1) because it is empirically found that cubic perovskites can form from 0.80 < *t* < 0.90 and 0.40 < *µ* < 0.90 [12, 20–22]. This implies that the large ionic radii of Pb (1.19 Å) and the halides (e.g., iodide 2.20 Å) limit the ionic radius of the monovalent A cation to 2.9 Å. Therefore, only two or three C–C or C–N bonds or inorganic cations such as Cs^+^ (1.88 Å) are expected to fit in the 3D hybrid perovskite structure [22].

One of the major advantages over traditional inorganic oxide is the low energy barrier to halide perovskite formation. A crystalline phase can be readily obtained by merely mixing and grinding the precursor salts at room temperature. Although this method suffers from a lack of precise experimental control, it exemplifies the ease with which the cations can diffuse into the inorganic framework. Typically, halide perovskites are synthesized via wet-chemistry routes, allowing mixing at a molecular level, and resulting in materials with a pure phase. By carefully controlling the reaction conditions (e.g., temperature, solvent, ligands), halide perovskites of various morphologies (0D to 3D) and sizes (ranging over six orders of magnitude) can be prepared. For example, CH_3_NH_3_PbBr_3_ single crystals with a size of 5 × 5 × 2 mm^3^ are obtained within a growth period of several hours by exploiting its lower solubility in solvents at elevated temperatures [23]. CH_3_NH_3_PbBr_3_ nanoparticles (NPs) [24–27], layered sheets [28, 29], and nanowires (NWs) [30, 31] could be prepared via tuning the synthesis strategy.

These examples briefly demonstrated the robust synthesis processes with which a wide scale of nanostructured perovskites can be synthesized. In recent years, nanomaterials have become more and more interesting, as the novel physical properties are only observed at the nanoscale range, in contrast to their larger-scale counterparts. Fine control over synthesis conditions (precursor concentration, reaction temperature, choice of ligands, etc.) could introduce new physical properties, such as quantum size effects [32] or anisotropic growth [33], to fulfill future application requirements.

## Synthesis and Fundamental Properties of Low**-**Dimensional Halide Perovskites

### Quantum Dots

QDs, combining unique optical and electrical properties and solution-processed advantages, have been studied intensively for decades. Here, we will cover the evolution of halide perovskite QDs and devices for light-emission applications. We will disclose the physical and chemical characteristics and analyze the rich diversity in composition and structure.

**Fig. 2:**
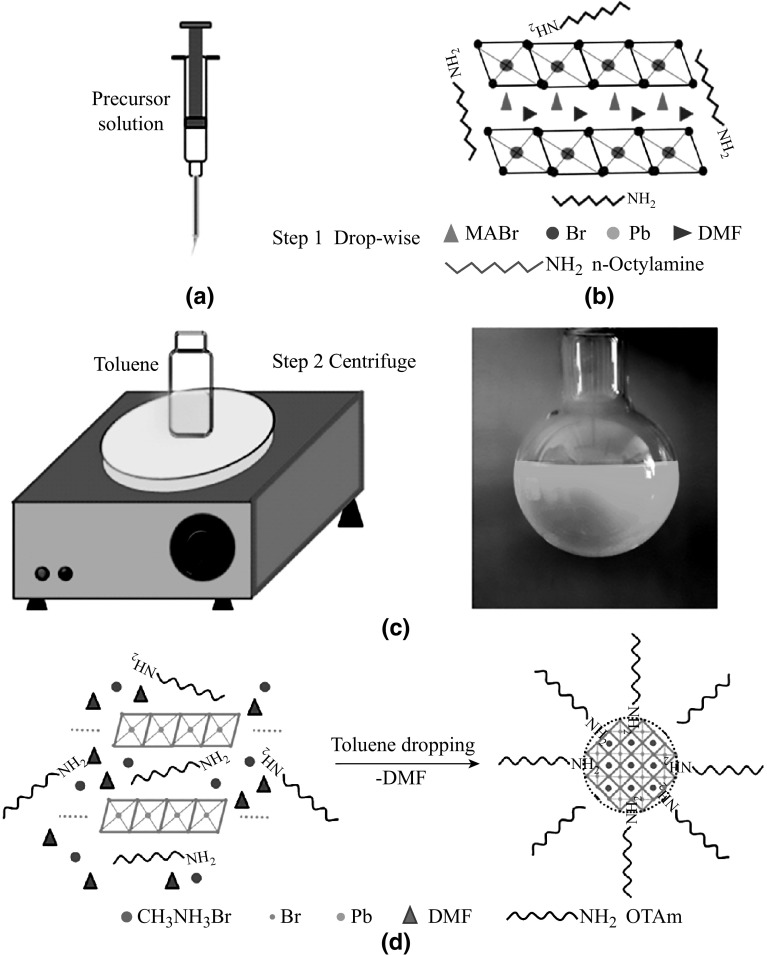
Synthesis of CH_3_NH_3_PbBr_3_ QDs. Schematic illustration of the reaction system (**a**) and precursors (**b**). **c** Optical image of colloidal CH_3_NH_3_PbBr_3_ solution. **d** Schematic illustration for the formation of the CH_3_NH_3_PbBr_3_ QDs. Adapted image reproduced with permission of Ref. [27, 34]

To date, most reports of hybrid perovskite NPs have utilized the distinct strategy of ligand-assisted reprecipitation (LARP) method. This method could fabricate high-luminescence and color-tunable colloidal CH_3_NH_3_PbI_3_ QDs with an absolute quantum yield (QY) up to 70% (Fig. 2). Polar solvents, capable of dissolving the inorganic lead and ammonium halide salts, are injected into a nonpolar “poor” solvent in the presence of coordinating ligands to stabilize the newly formed particles. It is noted that these syntheses are conducted at low temperature (<80 °C) [25–28, 32, 33, 35, 36]. Alternatively, CsPbX_3_ NPs could also be prepared using a hot injection method at higher temperatures ranging 140–200 °C under N_2_ atmosphere [37–40]. Here, the presence of oleic acid (OA) and oleylamine (OAm) ligands helps to inhibit crystal growth, passivate surface defects, and contribute to colloidal stability. This method has been successfully employed in the fabrication of high-quality inorganic semiconductor NPs [41]. One of the remarkable features of the hybrid perovskites is that no additional surface passivation is necessary to achieve high photoluminescence quantum yields (PLQYs), in contrast to traditional semiconductor QDs passivation, as the dangling bonds play a negligible role in the photoluminescence (PL) emission [37, 39].

The PLQY of QDs was gradually optimized by reaction and ligand selection. Utilizing long-chain ammonium bromide ligands, colloidal CH_3_NH_3_PbX_3_ cubic NPs (~6 nm) produced using the LARP method [28] exhibited bright-green PL emission at 527 nm, with a PLQY of approximately 20%. The increased reaction temperatures (~120 °C) yielding equal quantum efficiencies and PL emission wavelengths [42] showcased the rigidity and high reproducibility of the above method. The synthesis was further optimized by increasing the organic/inorganic precursor ratios. This could narrow the PL emission (FWHM = 30 nm at 520 nm) and significantly improve PLQY values to a new record of 83% [25]. Quantization effects were observed for 1.8- to 3.6-nm-sized CH_3_NH_3_PbBr_3_ NPs [27, 43]. The highest PLQY ~93% was recorded from the supernatant phase of centrifugation [25, 28].

Despite the high PLQY and colloidal stability of methylammonium halide NPs (>5 months of storage in air under dark conditions [25]), a major shortcoming arises from its instability in polar solvents. To overcome this limit, Vybronyi et al. synthesized CH_3_NH_3_PbX_3_ NPs without using polar solvents [44]. Although the precipitate displayed lower quantum efficiencies (25–50%) than previously reported CH_3_NH_3_PbBr_3_ NPs, it demonstrates an alternative synthesis route without the use of polar solvents. Other approaches involved the formation of PbS/CH_3_NH_3_PbX_3_ core–shell NPs via ligand-exchange reactions [45, 46]. Light emission associated with PbS/CdS NPs and PbS/CH_3_NH_3_PbI_3−*x*_Cl_*x*_ core–shell NPs has also been reported [47].

**Fig. 3:**
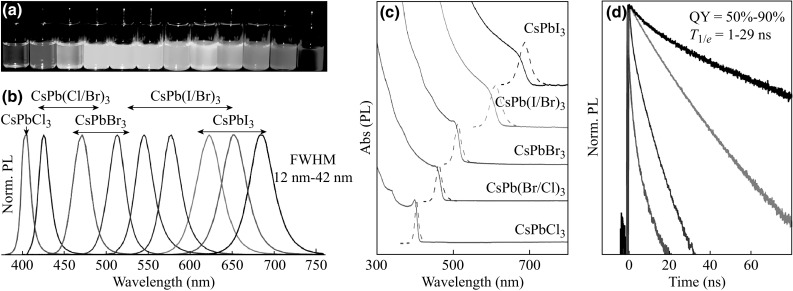
Colloidal CsPbX_3_ perovskite NCs (*X* = Cl, Br, I) exhibit size- and composition-tunable bandgaps covering the entire visible spectral region with narrow and bright emission: **a** Colloidal solutions in toluene under UV lamp (*λ* = 365 nm) excitation. **b** Representative PL spectra. **c** Typical optical absorption and PL spectra. **d** Time-resolved PL decays for all samples shown in **c** except CsPbCl_3_. Adapted image reproduced with permission of Ref. [37]

Beyond the methylammonium halide perovskite materials, color-tunable full-inorganic CsPbX_3_ perovskite NPs (4–25 nm in diameter) utilizing a hot injection method in a temperature range of 140–200 °C (Fig. 3) have been reported [37]. The resulting NPs exhibited high PLQY of 50–90% and narrow emission linewidths of 1242 nm. Owing to the large Bohr radius calculated for CsPbCl_3_ (5 nm), CsPbBr_3_ (7 nm), and CsPbI_3_ (12 nm), quantum confinement effects could be conveniently observed [37]. From transient absorption spectroscopy analysis, it was determined that the high PLQY arose from negligible electron–hole trapping pathways [48] and an average PL lifetime of 1–29 ns [34, 40, 48].

### Nanowires

The high absorption coefficient enables it as an amazing absorber, which was first applied in dye-sensitized solar cells (DSSCs) to replace organic dye by Kojima et al. [13], Horvath et al. [49]. The further investigation shows that they not only play the role of the light absorbers, but also can be viewed both as electron- and hole-transporting media, owing to their ambipolar charge transport character [50]. In perovskite film synthesis, a nonporous homogenous perovskite film must be deposited in order to avoid shunting in planar devices. However, films produced by conventional spin coating methods were found to be comprised of large CH_3_NH_3_PbI_3_ microwhiskers and many uncovered void areas [51]. Dendrite crystal growth implies that organolead iodide perovskite (OIP) exhibits preferential growth. Inspired by that phenomenon, the synthesis of OIP nanowires (NWs) was investigated and implemented in our group [52]. In addition to our work, Swiss scientists Endre Horvá et al. pioneered the synthesis of OIP NWs by a simple slip coating method during a similar period [53]. The above two works opened the new research fields of halide perovskite NWs. The key synthesis methods and novel properties are summarized in chronological order.

#### One-Step Evaporation-Induced Self-Assembly (EISA) Growth

For the one-step method, the precursor solution (MAI and PbI_2_ in DMF) was cast on substrates, and then, the evaporation of the solvent resulted in supersaturated crystal growth with the *c*-axis parallel to the surface at room temperature [54]. The low symmetry (tetragonal I4/mcm) of OIP and strong intermolecular interactions may allow a preferred growth into NW morphology [55, 56]. For a high substrate temperature (>120 °C), it tended to grow homogeneously into NPs and suppress the preferential growth. Subsequent thermal annealing (~80 °C) can help to convert the NWs precursor into perovskite NWs.

**Fig. 4:**
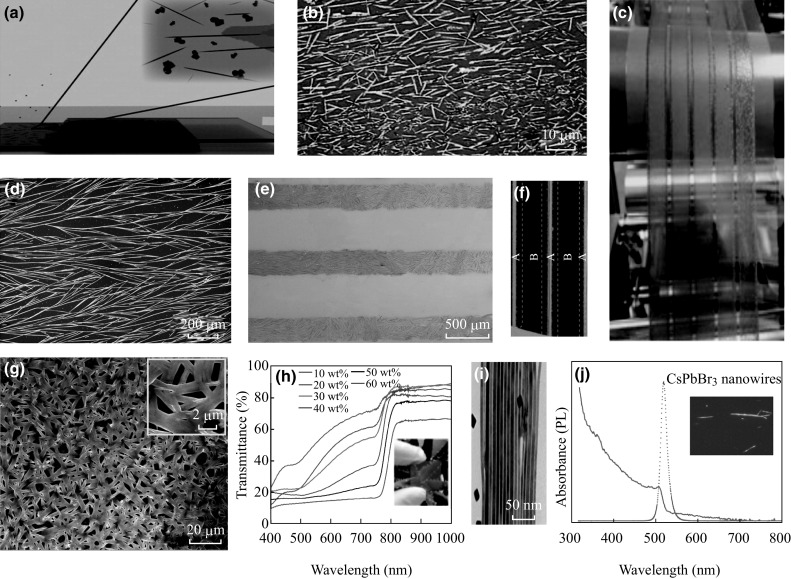
One-step growth of perovskite NWs. **a**, **b** Slip coating fabrication process of filiform halide perovskite and their optical image. **c**, **f** Roll-to-roll printing for perovskite NWs thin film. **d** EISA method to fabricate aligned perovskite NWs. **e** Selective area deposition of perovskite NWs. **g** SEM image of NWs webs. **f** Transmittance spectra of NWs webs evolved with the precursor concentrations. *Inset* shows a photograph of semitransparent NWs networks from 30 wt% concentration. **h** Transmittance evolved with OTP precusor concentrations. **i**, **j** Full-inorganic CsPbBr_3_ NWs synthesized by one-step method and their typical optical absorption and PL spectra (**j**). Adapted image reproduced with permission of Ref. [49, 52, 57–59]

In most cases, self-assembled NWs by solution methods tend to distribute in a macroscopically random manner on substrates. The uniform distribution rather than NW bundles was implemented by UV–ozone treatment, owing to the improvement in surface DMF infiltration (Fig. 4d). The selective area growth and patterning of perovskite NWs were also implemented by UV–ozone treatment assisted by a shadow mask in our first CH_3_NH_3_PbI_3_ NW work [58] (Fig. 4e). In addition, Endre Horvá et al. utilized two glass microscope slides sandwiching saturated MAPbI_3_ DMF solution to shape an ultrathin MAPbI_3_ precursor solution in order to control the NW distribution. Combined variations of solvent concentration, temperature, fluidphilicity/phobicity, and sliding speed were utilized to control the crystallization kinetics, yielding tunable NW width, height, and length (Fig. 4a, b).

Our further work created a network-like NW web, which could satisfy the cross-linking and uniform NW distribution requirements to overcome the obstacles to NW applications [52]. In contrast to our previous drop-casting style, we adopt a spin coating method with suitable rotary speed and an annealing technique to obtain perovskite NW webs (Fig. 4g). All the NWs welded to each other without opening the end. The transparency of NWs webs could be facilely tuned by the precursor concentrations (Fig. 4h). The low-temperature fabrication process and web geometry promise high application potential in transparent and flexible optoelectronics. The usual one-step growth methods often achieve large diameter NWs over 200 nm. Yang and coworkers added a surfactant solvent into the precursor to tune the NW crystallization kinetics [59] (Fig. 4i).

The application of low-dimensional NWs needs large-scale growth to meet the wide application fields. The blade coating method enables the synthesis of aligned single-crystalline OIP microwire (MW) arrays in terms of high yield and wafer size capability. Thus, Jie et al. first applied the doctor blade coating technique for large-scale and aligned NW growth [60]. It is well known that the highest efficiency deposition technique is printing. The low deposition temperature and one-step solution method promise potential applicability by printing methods. Recently, Yang et al. developed a large-scale roll-to-roll microgravure printing technique for perovskite NWs synthesis [61] (Fig. 4f). By systematic deposition recipe optimization, perovskite NW thin film was deposited on PET substrates (Fig. 4c).

#### Two-Step Method Growth

**Fig. 5:**
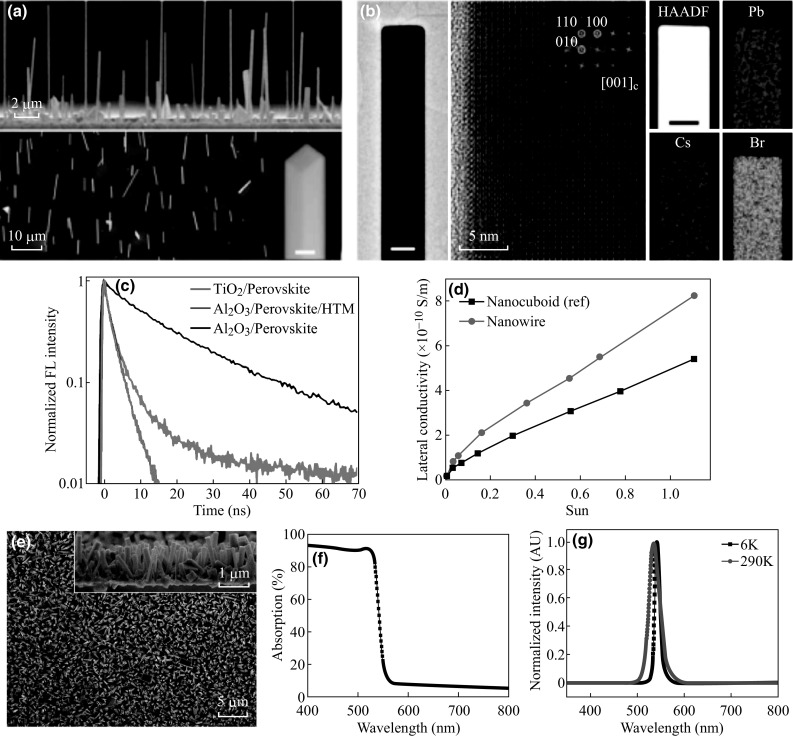
Two-step method for perovskite NW growth. **a** SEM images of vertically aligned CsPbBr_3_ NWs with a rectangular cross section. **b** HRTEM and FFT images of a CsPbBr_3_ NWs. **c** Fluorescence decay kinetics of MAPbI_3_ NWs. **d** Conductivity improvement in the in-plane perovskite NWs. **e** SEM image of CH_3_NH_3_PbBr_3_ nanorod array. **f, g** Typical absorption and PL spectra of CH_3_NH_3_PbBr_3_ nanorod arrays. Adapted image reproduced with permission of Ref. [30, 63]

Similar to perovskite thin-film fabrication, researchers developed a two-step method in order to face the specific requirements. Without using a novel technique such as electrospinning, MAPbI_3_ NW film was conveniently formed by two-step spin coating technology. They simply coated the PbI_2_ layer with an isopropanol solution of MAI in the presence of a small amount of polar aprotic solvent [30]. The NW diameters ranged from 30 to 200 nm, much smaller than those obtained from a one-step solution method. The locally dissolved PbI_2_ may serve as a preferential site for reacting with MAI to grow a 1D structure, like a liquid catalyst cluster model [62]. Time-resolved fluorescence spectroscopy confirmed that charge separation at ETL/perovskite was faster for 1D NWs than for 3D nanocubes, because of the lager surface area of the former structures (Fig. 5c). The conductivity of NW film was enhanced by a factor 1.3–1.6, indicating better connectivity pathways and apparently increased mobility (Fig. 5d).

Similarly, Yang’s group replaced the second spin coating step with immersion in CH_3_NH_3_Br IPA solution [30]. The lead acetate substrate could support vertical perovskite nanorod arrays (Fig. 5e). The NW absorption onset was at approximately 530 nm with a bandgap of 2.3 eV [64] (Fig. 5f). The room-temperature PL of the CH_3_NH_3_PbBr_3_ NWs peaked at 534 nm with a narrow FWHM of 26 nm under 325-nm HeCd laser excitation (Fig. 5g). The temperature-dependent phases of the CH_3_NH_3_PbBr_3_ NWs underwent multiple phase transitions at 236.3 (to a tetragonal phase), 154.0 (to another tetragonal phase), and 148.8 K (to an orthorhombic phase), which may account for the observed redshift [65]. In comparison with bulk crystal PL, the CH_3_NH_3_PbBr_3_ nanorod arrays had a 13% contribution of the fast component to the steady-state PL, which indicated the high quality of the single-crystalline nanorods.

#### Chemical Vapor Transport (CVT) Method

This instrument is the same as a tube furnace for traditional chemical vapor deposition [66]. The reaction sources of PbX_2_ and CsX (*X* = Cl, Br, or I) powders were placed inside a quartz tube reactor [66]. The Si substrate was positioned at a distance from the source. The temperatures of the powder sources and Si substrate were set at 570–600 and 350–380 °C, respectively. The inert carrier gas was utilized to transport the source vapor for deposition on the Si substrate (Fig. 5a, b). Apart from the above-mentioned research, Yi-bing Cheng et al. also utilized the CVT method to synthesize perovskite MWs [67].

#### Template Method

**Fig. 6:**
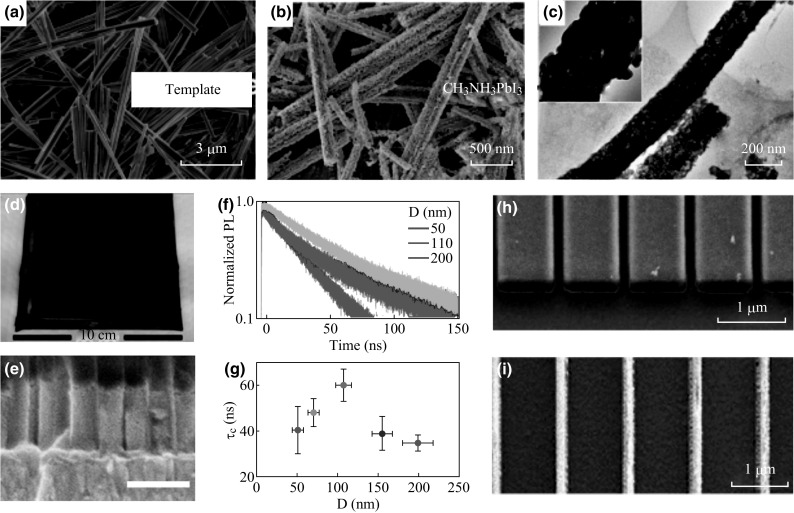
Template method for perovskite NWs: Pb-containing precursor template **a** method for perovskite NWs (**b**, **c**). AAO template for perovskite NWs. **d** Photograph of a ~9 × 9 cm^2^ NW arrays. **e** Cross-sectional SEM images of perovskite NWs. *Scale bar* is 500 nm. **f** Average *τ*_c_ for different diameter NWs. **g** Extension of carrier lifetime as the decrease in NW diameter. **h, i** Nanofluidic channel template growth. Adapted image reproduced with permission of Ref. [68, 70, 71]

According to the lead ionic precursor NWs and the later morphology-retaining reaction, Zhang’s group introduced a new lead NW template as the lead source [68]. The reaction between Pb(NO_3_)_2_ and l-cysteine aqueous solution with the assistance of ethanolamine could yield the Pb precursor NW templates [69]. The solid hybrid Pb-containing NWs further transformed into porous CH_3_NH_3_PbBr_3_ perovskite NWs by the addition of CH_3_NH_3_Br and HBr 2-propanol solution (Fig. 6a–c).

The traditional universal templates, anodized aluminum oxide (AAO) templates, were also applied for the synthesis of perovskite NWs [70]. Oriented cylindrical nanopores constructed the uniform vertical perovskite nanowire array templates. This method combined the AAO template and perovskite one-step solution method. Perovskite precursor DMF solution filled into the pores of AAO. The following thermal annealing led to perovskite recrystallization and NW formation at the bottoms of the pores (Fig. 6d, e). The advantage of the present AAO template method is the fine-tuning of the NW dimensions (diameter and length) by controlling the AAO anodizing recipes [66, 72–74].

Different from the vertical templates, traditional planar electronic fabrication techniques such as photolithography, electron beam lithography could help to fabricate the 1D channel as the planar growth template. The perovskite NWs were fabricated with the assistance of open nanofluidic channel templates [30] (Fig. 6h, i). The synthesis could be guided and visualized in real time and underwent a metastable solvatomorph formation in polar aprotic solvents. The superior advantages were the precise controlling of sizes, cross-sectional shapes, aspect ratios, and orientations, which had not been achieved by other deposition methods.

### Two-Dimensional Perovskite

Two-dimensional (2D) perovskites such as nanosheets, nanoplatelets, and microdisks (MDs) have recently shown high PLQY [75–77]. These 2D perovskites are promising candidates for a variety of applications in nanoelectronics, nanophotonics, and photovoltaics [32, 78]. For instance, Zhao and Zhu [79] prepared MAPbI_2_Br nanosheets with a 1.8-eV bandgap through a thermal decomposition process from a precursor containing PbI_2_, MABr, and MACl. The planar solar cells based on the compact layer of MAPbI_2_Br nanosheets achieved a PCE of ~10%.

**Fig. 7:**
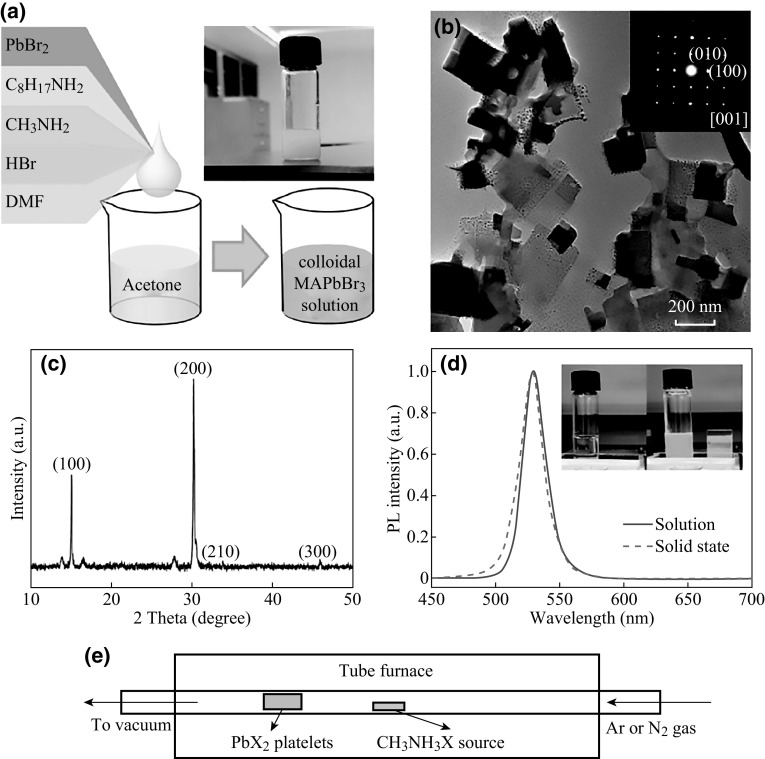
**a** Single-crystalline organometal halide perovskite nanoplatelets were synthesized by a one-pot method. *Inset* is the photograph of the achieved colloidal perovskite nanoplatelet solution. **b** TEM images and selected area diffraction pattern of MAPbBr_3_ nanoplatelets. **c** X-ray diffraction of MAPbBr_3_ nanoplatelet film. **d** PL spectra of MAPbBr_3_ nanoplatelets in toluene solution and solid state. *Inset*, the optical images of MAPbBr_3_ nanoplatelets in toluene solution and in solid-state thin film under ambient light (*left*) and UV irradiation (*right*). **e** Schematic drawing of the nanoplatelet synthesis setup using a home-built vapor transport system. Adapted image reproduced with permission of Ref. [76]

**Fig. 8:**
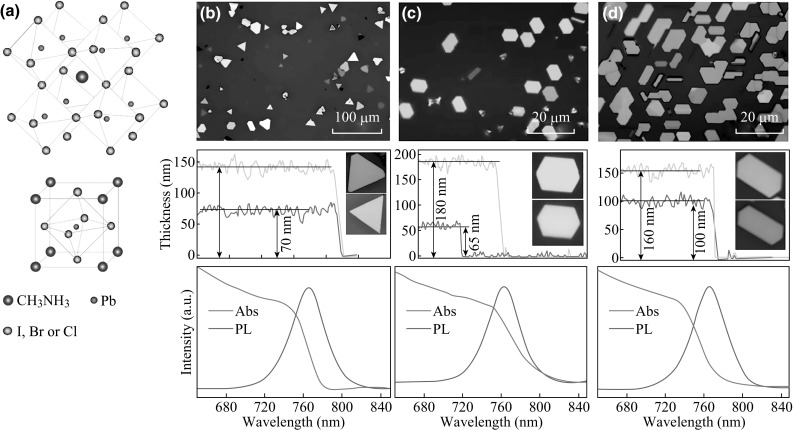
Chemical vapor-deposited methylammonium lead halide perovskite nanoplatelets. **a** Schematic figure of crystal structures of CH_3_NH_3_PbI_3−*a*_X_*a*_ (*X* = I, Br, Cl). Morphological and electronic band edge characterizations of CH_3_NH_3_PbI_3_ (**b**), CH_3_NH_3_PbI_3−*a*_Br_*a*_ (**c**), and CH_3_NH_3_PbI_3−*a*_Cl_*a*_ (**d**) nanoplatelets. Adapted image reproduced with permission of Ref. [24]

Xiong’s group successfully grew well-defined polygonal CH_3_NH_3_PbX_3_ (*X* = Cl, Br, I) nanoplatelets by a chemical vapor method [24, 80]. As shown in Fig. 7, PbX_2_ platelets were first prepared on muscovite mica using van der Waals epitaxy in a vapor transport chemical deposition system. Subsequently, these PbX_2_ platelets were placed downstream in a quartz tube to react with MAX vapor by a gas–solid heterophase reaction for conversion into perovskite nanoplates. Figure 8 shows the crystal structure and morphologies of perovskite nanoplatelets. Interestingly, the thickness of PbI_2_ correlated with OIP platelets by a factor of 1.81, which was in good agreement with their lattice constant ratio along the *c*-axis. This work offers a reliable method to control the thickness of perovskite platelets. Later, the same group readily applied these perovskite nanoplatelets to fabricate near-infrared solid-state lasers, which exhibited low thresholds and wide mode tunability [24].

Recently, Liao et al. [81] fabricated single-crystalline CH_3_NH_3_PbBr_3_ square MDs-based microlasers by using a one-step solution self-assembly method. That approach was similar to anti-solvent vapor-assisted crystallization method [82]. The obtained square MDs had smooth outer surfaces and sharp edges and displayed an absorption peak at 535 nm and an emission peak at 545 nm. Their four side faces constituted a built-in whispering-gallery mode microresonator with a quality factor as high as 430. By partial replacement of Br with Cl, the lasing wavelength can be effectively tuned in the green-light range from 525 to 557 nm.

**Fig. 9:**
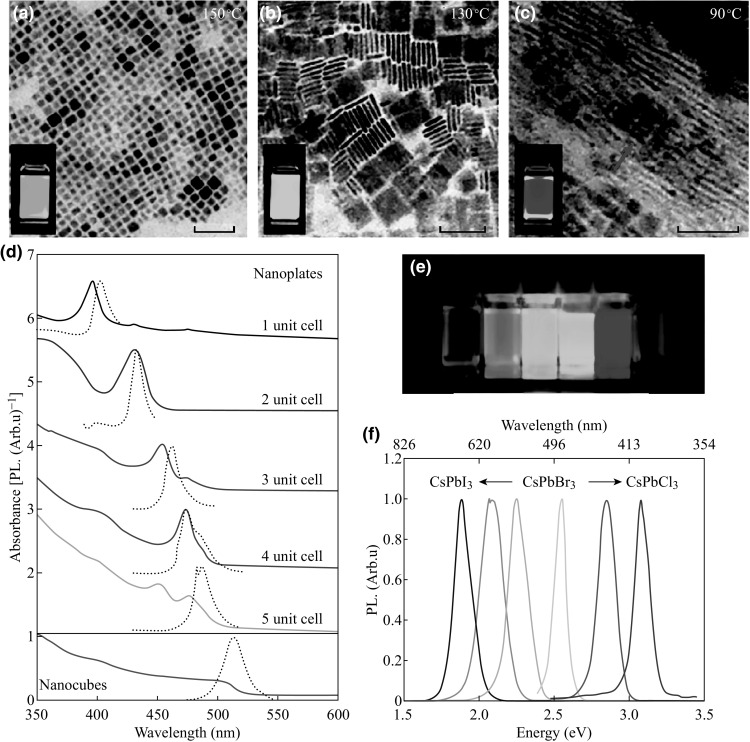
Reaction temperature influence on colloidal CsPbBr_3_ synthesis. 8- to 10-nm nanocubes were formed at 150 °C (**a**), 20-nm nanoplates from 130 °C (**b**) reaction and 90 °C, **c** reaction yielded several hundred nanometer scale lamellar nanostructures. *Scale bar* is 50 nm. **d** Absorption (*solid lines*) and emission (*dashed lines*) spectra of NPLs and nanocubes for comparison. **e** Colloidal solution of anion-exchanged NPLs in hexane under UV illumination (*λ* = 365 nm). **f** Inorganic perovskite PL peaks evolved with their anions. Adapted image reproduced with permission of Ref. [75]

In a more recent study, 2D MAPbBr_3_ nanoplatelets with nearly single unit cell thickness and submicron lateral dimensions were prepared by a colloidal synthesis method [29]. Those 2D nanoplatelets exhibited a single and sharp excitonic absorption featured at 431 nm, which blueshifted by 0.5 eV from that of the 3D bulk perovskite phase. This large blueshift was a clear evidence of one-dimensional quantum confinement. A similar colloidal synthetic method was used for full-inorganic CsPbBr_3_ nanoplatelets (Fig. 9), which exhibited narrow PL and strong excitonic absorption [83]. Recently, Yu et al. demonstrated that the crystallinity of monoclinic CsPbBr_3_ was much lower than that of tetragonal CsPb_2_Br_5_, and thus, they reported a new type of highly luminescent perovskite-related pure tetragonal CsPb_2_Br_5_ nanoplatelets synthesized by a facile precipitation reaction. Moreover, a new kind of pure phase perovskite nanosheets can be obtained by utilizing cation exchange and anion exchange [84]. In the tetragonal CsPb_2_Br_5_ structure, one layer of Cs ions is sandwiched between two layers of Pb–Br coordination polyhedrons, which have an obviously different crystal structure from the CsPbBr_3_ perovskite. Jiang et al. synthesized CsPb_2_Br_5_ nanosheets by using a solution-phase method. They found that the tetragonal CsPb_2_Br_5_ nanosheets were formed via an oriented attachment of CsPbBr_3_ nanocubes with an orthorhombic structure [85]. Jang et al. [86] synthesized MAPbBr_3_ nanoplates using octylamine as the capping ligand. The composition of nanoplates was tunable by a simple halide exchange reaction. Furthermore, Yang et al. reported the solution growth of atomically thin, uniform, and square-shaped 2D hybrid perovskites of (C_4_H_9_NH_3_)_2_PbBr_4_ [87]. Different from conventional 2D materials, the (C_4_H_9_NH_3_)_2_PbBr_4_ sheet exhibited an unusual lattice constant expansion, which led to a slightly shifted band edge emission relative to the bulk counterpart. Those 2D crystals also displayed high PL quantum efficiency and color tunability through halide substitution and thickness variation.

It is interesting to note that perovskite nanosheets were surprisingly obtained from electrospraying the precursor solution into a mixed bath of toluene (as anti-solvent) and oleylamine (for intercalation) [88]. Recently, single crystals of OIP nanoplates with well-defined facets were grown via a dissolution–recrystallization path from PbI_2_ (or PbAc_2_) films [89]. These 2D perovskite nanostructures displayed strong room-temperature PL and long carrier lifetime.

## Low-Dimensional Perovskite Optoelectronic Applications

### Light-Emitting Diodes

The key parameter for high-performance light-emitting materials is the PLQY, defined as the ratio of emitted to absorbed photons. On the LED level, the radiant efficiency (RE) or wall plug efficiency (WPE) is the electrical-to-optical energy conversion efficiency described as:

$${\text{RE}}\left( {{\text{or}}\;{\text{WPE}}} \right) = {\text{EQE}} \times {\text{LE}} = \eta_{\text{injection}} \times {\text{IQE}} \times \eta_{\text{extraction}} \times \left( {L/IV} \right),$$

where the external quantum efficiency (EQE) is defined as the ratio of emitted photons to the number of electrons injected into the device and the luminous efficacy (LE) is the ratio of emitted photons to the energy injected by the source (*IV*). The ultralow amplified spontaneous emission (ASE) thresholds of organometallic halide perovskites provide a strong impetus for light-emission applications.

**Fig. 10:**
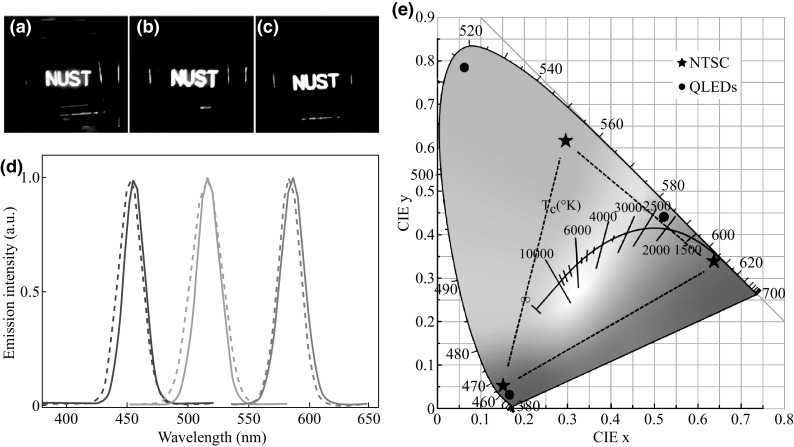
**a**–**c** Emissions of QLED devices utilizing different QD sizes. **d** The EL (*solid line*) and the PL spectra (*dashed line*) of samples shown in (**a–c**). **e** CIE coordinates of the three color QLEDs (*circular*) compared to the NTSC color standards (*star*). Adapted image reproduced with permission of Ref. [90]

The extraordinary enhancement of the PLQY in the past 2 years has now placed perovskites on a par with the best-in-class solution-processed semiconductors. Different strategies, such as synthesis of low-dimensional layered perovskites, or increasing spatial confinement, have been applied to maximize PLQYs. The high PLQYs combined with the compositional flexibility of these materials render perovskites robust technological candidates. They are distinguished by: (1) high color purity (FWHM ~20 nm) [37] (Fig. 10), irrespective of the crystallite size [32, 91]; (2) bandgap tunability covering the entire spectrum of visible light in layered perovskites [92, 93]; and (3) low to moderate ionization energy to form stable functional interfaces [94].

**Fig. 11:**
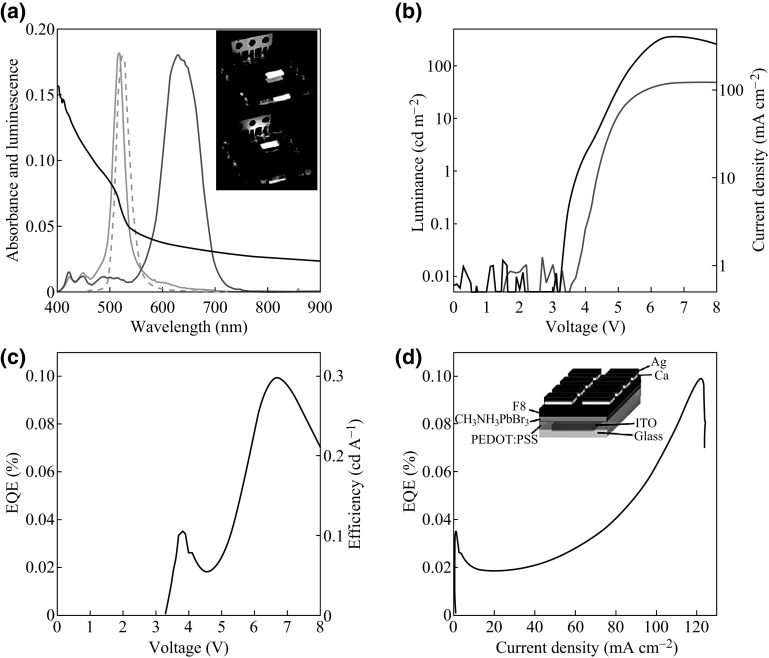
Device characteristics of visible PeLEDs. **a** Absorption (*black*), normalized EL (*solid line in green*), and PL (*dashed line in green*) spectra of CH_3_NH_3_PbBr_3_ perovskite. EL spectrum of mixed halide perovskite is shown in *red*. **b** Luminance (*black*) and current density (*red*) versus voltage characteristics of the *green* PeLED. **c** EQE versus voltage characteristics of the *green* PeLED. **d** EQE versus current density of the *green* PeLED. Adapted image reproduced with permission of Ref. [96]. (Color figure online)

Organic–inorganic lead-based perovskites were first investigated for light-emitting applications before they were used as absorbers in DSSC devices. Perovskite LEDs were first demonstrated in the 1990s using organic–inorganic perovskites with layered two-dimensional structures. However, electroluminescence from radiative recombination of injected electrons and holes was only observed at liquid nitrogen temperatures. Recently, perovskite light-emitting diodes (PeLEDs) of the low-dimensional metal halide perovskites MAPbI_3−*x*_Cl_*x*_, MAPbBr_3_, and MAPbI_3−*x*_Br_*x*_ have been demonstrated. These devices operated at room temperature under substantial current densities and brightness. The internal quantum efficiencies reached 3.4%, and the emissions were located at near-infrared and green (Fig. 11). Comparable emission has also been demonstrated in an inverted structure by tuning the work function of the conventional PEDOT:PSS hole injection layer. However, the above two devices require high charge densities for efficient radiative recombination. Enhanced device emission and quantum efficiency would be obtained by removing or filling material trap states and optimizing film quality, similar to halide perovskite solar cells.

**Fig. 12:**
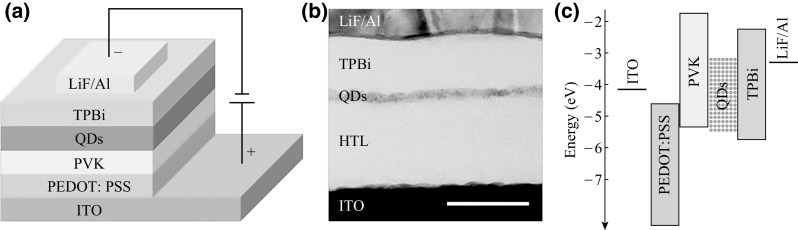
Illustration of multilayer PeLED device. **a** Device structure. **b** Cross-sectional TEM image of multiple layers with distinct contrast. *Scale bar* is 50 nm. **c** Flat-band energy level diagram. Adapted image reproduced with permission of Ref. [90]

In addition to possessing maximal external luminescence, a good light-emitting device requires efficient carrier injection and radiative recombination. In particular, balancing the electron and hole injection by tailoring the interfaces helps to focus the radiative recombination within the desired emitter layer. In this respect, metal halide perovskites can meet the charge transport and charge injection requirements with high carrier mobility and optimum interface formation with most employed contacts. In PeLEDs, the emitter layer may comprise 3D, layered, or nanostructured perovskites sandwiched between electron and hole transport layers (Fig. 12). The EQE of PeLEDs has rapidly progressed from less than 1% to over 8% in fewer than 2 years. In addition, the CE and luminance are approaching the QLED and OLED top ranges. Tae-Woo Lee and coworkers used an organic small molecule, 2,2′,2″-(1,3,5-benzinetriyl)-tris(1-phenyl-1-H-benzimidazole), as an additive to further reduce perovskite grain size. The perovskite exciton diffusion length was decreased by spatial confinement in uniform MAPbBr3 nanograins. Simultaneously, they prevented the formation of metallic lead (Pb) atoms that cause strong exciton quenching through a small increase in methylammonium bromide (MABr) molar proportion. They eventually boosted the current efficiency (CE) of PeLEDs with a simple bilayer structure to 42.9 cd A^-1^ [95]. The representative results of PeLEDs are summarized in Table 1. Further breakthroughs in PeLED performance mainly include material design, optimized charge injection layers, and emission tunability to yield high color quality, purity, and white-light emission.

**Table 1:** Overview of selected representative results of PeLED [36, 76, 88–93, 95]

Perovskite emitter	Morphology	Device architecture	EQE (%)	CE (cd A^2^)	L_ma*x*_ (cd m^−2^)	Vr (V)	Publication date (month-year)
CH_3_NH_3_PbBr_3_	Thin film	ITO/PEDOT:PSS/Pe/F8/Ca/Ag	0.1	0.3	364	3.3	08-2014
CH_3_NH_3_PbBr_3_	Thin film	ITO/Buf HIL/Pe/TPBI/LiF/Al	0.125	0.57	417	~4	11-2014
CH_3_NH_3_PbBr_3_	Thin film	ITO/PEDOT:PSS/TPD/Pe/Ag	6.5 **×** 10^−3^	~1.8 **×** 10^−2^	21	4	01-2015
CH_3_NH_3_Pb_3−*x*_Br_*x*_	Thin film	ITO/PEDOT:PSS/TPD/Pe/Ag	1.1 **×** 10^−3^	n.r	n.r	n.r	01-2015
CH_3_NH_3_PbBr_3_	Thin film	ITO/PEDOT:PSS/Pe/ZnO/Ca/Ag	n.r.	~21	~550	2	02-2015
CH_3_NH_3_PbBr_3_	Thin film	ITO/PEDOT:PSS/Pe-PIP/F8/Ca/Ag	1.2	n.r	200	n.r	04-2015
CH_3_NH_3_PbBr_3_	Thin film	ITO/ZnO-PEI/Pe/TFB/MoO_*x*_/Au	0.8	n.r	20,000	2.8	04-2015
CH_3_NH_3_Pb_3−*x*_Cl_*x*_(red)	Thin film	ITO/ZnO-PEI/Pe/TFB/MoO_*x*_/Au	3.5	n.r	n.r	2.2	05-2015
CH_3_NH_3_Pb_3−*x*_Cl_*x*_(red)	Thin film	FTO/TiO_2_/Pe/Spiro-MeTAD/Au	0.48	n.r	n.r	1.5	05-2015
CH_3_NH_3_PbBr_3_	Thin film	ITO/c-TiO_2_/EA/Pe/SPB-02T/MoO_*x*_/Au	0.051	0.22	545	n.r	07-2015
CH_3_NH_3_Pb_3−*x*_Cl_*x*_	Thin film	ITO/Mg-ZnO/Pe/CBP/MoO_*x*_/Au	0.1	n.r	n.r	2.2	08-2015
CH_3_NH_3_PbBr_3_	Thin film	ITO/Pe-PEO/In-Ga	0.083	0.38	4064	2.9	10-2015
CsPbBr_3_	QD	ITO/PEDOT:PSS/PVK/Pe/TPBI/LiF-Al	0.12	0.43	946	4.2	10-2015
CsPbBr_3_	Thin film	ITO/PEDOT:PSS/Pe/F8/Ca/Ag	0.008	0.035	407	3	11-2015
CH_3_NH_3_PbBr_3_	Thin film	ITO/PEDOT:PSS/Pe/SPB-02T/LiF/Al	0.1	0.43	3490	2.4	11-2015
CH_3_NH_3_PbBr_3_	NPLs	ITO/PEDOT:PSS/Pe/PVK:PBD/BCP/LiF/Al	0.48	n.r	10590	3.8	11-2015
CH_3_NH_3_PbBr_3_	Thin film	ITO/PEDOT:PSS/Pe(6%HBr)/SPB-02T/LiF/Ag	0.2	0.43	3490	4.3	11-2015
CH_3_NH_3_PbBr_3_	Thin film	Glass/SOCP/Pe/TPBI/LiF-Al	8.53	42.9	~15,000	4	12-2015
CH_3_NH_3_PbBr_3_	Printed thin film	ITO/Pe-PEO/Ag NWs	1.1	4.91	21,014	2.6	12-2015

All perovskites display green emission unless specially stating

Pe, perovskite; ITO, In-doped SnO_2_; PEDOT:PSS, poly(3,4-ethylenedioxythiophene):polystyrene sulfonate; F8, poly(9,9-dioctylfluorene); Buff-HIL, buffered hole injection layer; TPBI, 2,2′,2″-(1,3,5-benzinetriyl)-tris(1-phenyl-1-H-benzimidazole); TPD, *N*,*N*′-bis(3- methylphenyl)-*N*,*N*′-diphenylbenzidine); PIP, polyimide polymer; PEI, poly(ethylenimine), TFB, poly(9,9-dioctylfluorene-co–*N*-(4-butylphenyl)diphenylamine); EA, ethanolamine; SPB-02T, blue copolymer, Merck Co.; BCP, bathocuproine; PEO, poly(ethyleneoxide); PVK, poly(9-vinlycarbazole); PBD, 2-(4-biphenylyl)-5-phenyl-1,3,4- oxadiazole; n.r, Not reported

### Solar Cells

**Fig. 13:**
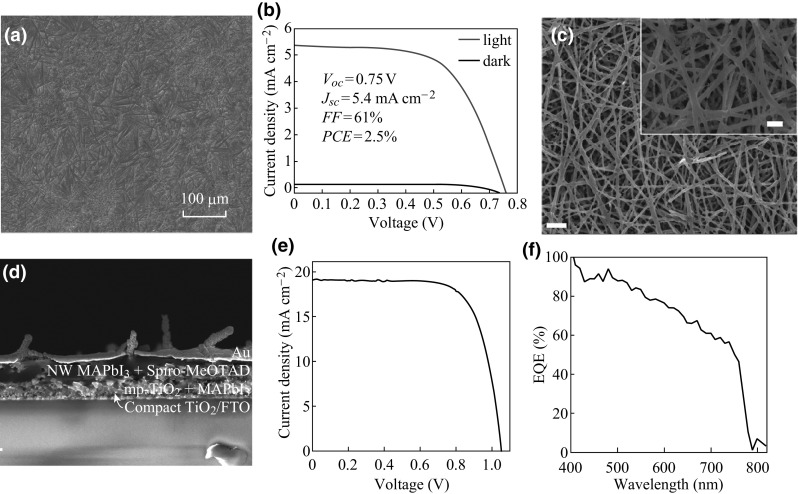
Perovskite NW solar cells: **a**, **b** SEM image of NWs from a one-step method and its corresponding *J*–*V* curve. Two-step-method-grown NWs and corresponding device performances. **c**, **d** Top-view and cross section of NWs films. **e**, **f** *J*–*V* and IPCE curves of NW solar cells. The *scale bar* is 1 μm. Adapted image reproduced with permission of Ref. [52, 63]

Perovskite NPs were originally used as sensitizers in DSSCs. Our group first reported perovskite NW solar cells utilizing the same device structure as thin-film solar cells (Fig. 13a, b) [52]. Compared to the contemporary champion efficiency (∼19.3%) of OIP thin-film solar cells [4, 78, 97], the obtained *J*_sc_ values were much lower because of the thicker film required to avoid shorting of the inter-NWs void. However, for specific fields, the non-full fill of substrate by NWs may find suitable applications such as semitransparent solar cells, building-integrated photovoltaics. The elementary results were greatly improved by the group of Nam-Gyu Park [63]. In order to avoid the void-induced shorting effect, they developed a two-step method to grow denser and smaller-diameter NWs as the absorber (Fig. 13c, d). Perovskite NWs with a mean diameter of 100 nm showed faster carrier separation in the presence of the hole-transporting layer and higher lateral conductivity than their 3D counterparts. The best performing device exhibited a photocurrent density of 19.12 Ma cm^−2^, voltage of 1.052 V, and fill factor of 0.721, leading to a PCE of 14.71% with small *I*–*V* hysteresis (Fig. 13e, f).

There are many reviews discussing about halide perovskite thin-film solar cells. Thus, we only list the key breakthroughs in chronological order. Miyasaka and coworkers were the first to report CH_3_NH_3_PbBr_3_ solar cells with a PCE of 2.2% in 2006 [98]. In 2009, they improved the PCE to 3.8% by replacing bromine with iodine. Subsequently, Park and colleagues optimized a titania surface and substituted DMF solvent by γ-butyrolactone, yielding an efficiency of triiodide cells to 6.5% in 2011 [14]. Unfortunately, the device stability had not significantly improved. Until 2012, Kanatzidis and coworkers utilized the *p*-type solution-processable perovskite fluorine-doped CsSnI_3_ as a solid HTL in a solid-state DSSC [99]. This was the first time that a perovskite material has been used as the HTL with efficiencies up to 10.2%. The real breakthrough of perovskite stability was obtained by the Gratzel and Park groups [5]. They utilized MAPbI_3_ as a light harvester combined with the solid hole conductor 2,2′,7,7′, tetrakis(*N*,*N*-dimethoxy-phenyl-amine)-9,9′-spirobifluorene (spiro-MeOTAD) on mesoporous TiO_2_ leading to a PCE of 9.7% and dramatically improving the device stability compared to CH_3_NH_3_PbI_3_-sensitized liquid junction cells. From the above-mentioned research [5, 99], it can be concluded that OIP had the excellent capability to achieve ambipolar charge transport. At the same time, a significant study reported by Seok, M. Gratzel, and coworkers [100] boosted the PCE to 12%.

**Fig. 14:**
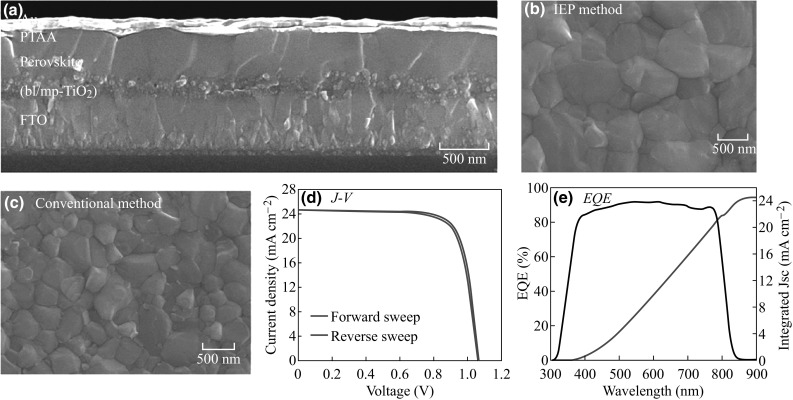
SEM, *J*–*V*, and EQE measurements for perovskite solar cells. **a** Cross-sectional SEM image of the device. The comparison of SEM surface images of FAPbI_3_-based layer formed on mp-TiO_2_ by IEP (**b**) and conventional method (**c**). **d** *J*–*V* curves of best device measured in reverse and forward modes, and **e** EQE spectra for best device and integrated *J*_SC_. Adapted image reproduced with permission of Ref. [101]

In the middle of 2013, there were two important reports published in the journal Nature, which accelerated the study of perovskite solar cells to a new level [4, 16]. Both of them had achieved the PCE over 15% by using new, modified perovskite processing methods. One defined as “sequential deposition” produces a device structure of FTO/cp-TiO_2_/mp-TiO_2_/MAPbI_3_/spiro-OMeTAD/Au [102] and the other developed by H. Snaith and coworkers deposited a high-quality MAPbI_3−*x*_Cl_*x*_ film via dual-source vacuum deposition in a planar heterojunction (PHJ) perovskite solar cell, as shown in Fig. 14. This FTO/cp-TiO_2_/MAPbI_3−*x*_Cl_*x*_/spiro-OMeTAD/Ag-based device achieved a PCE 15.4% [4]. Up to now, to the best of our knowledge, Seok and coworkers maintain the record certified PCE of 22.1% (Fig. 14). Simultaneously, all-inorganic perovskite solar cells have been reported. Liu and coworkers for the first time discovered how to make CsPbBr_3_ perovskite solar cells out of QDs and achieved 6.7% conversion efficiency. Recently, Joseph M. Luther and coworkers implemented a low-temperature synthesis of α-CsPbI_3_ perovskite. The highest PCE of all-inorganic perovskite solar cells reached 10.77% [103].

**Fig. 15:**
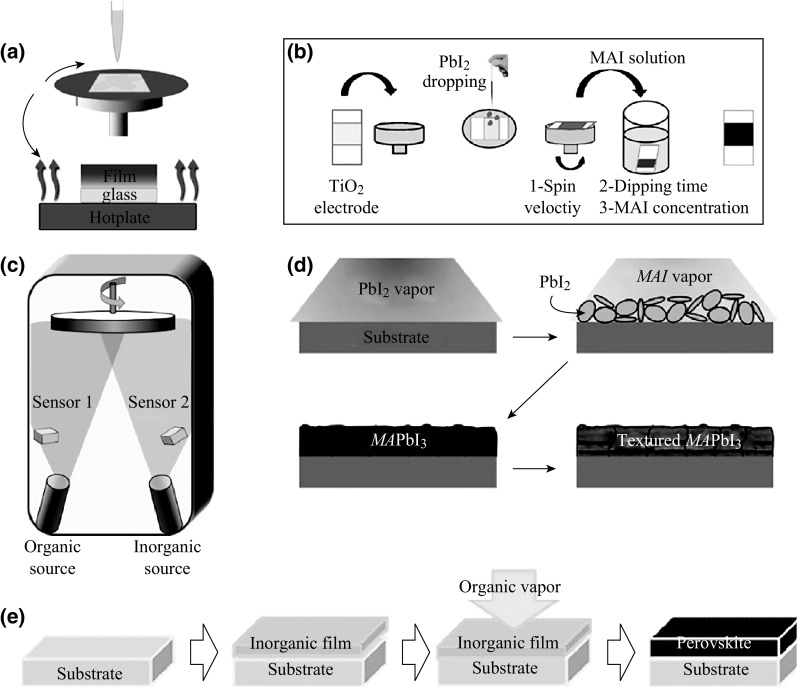
**a** One-step deposition. **b** Two-step sequential deposition. **c** Dual-source coevaporation. **d** Sequential vapor deposition. **e** Vapor-assisted solution growth process. Adapted image reproduced with permission of Ref. [18, 105–107]

As the PCEs of perovskite solar cells are very sensitive to the morphology, composition, uniformity, and solution process, among others, the fabrication of perovskite absorbers must be accurately controlled. The main methods (Fig. 15) include one-step deposition [104] and two-step sequential deposition [4] methods based on solution processing, vacuum deposition [16, 105], and vapor-assisted solution processes [18]. In addition, a fast deposition crystallization (FDC) procedure was developed to yield highly uniform perovskite film consisting of microsized crystals, as illustrated in Fig. 15.

### Photodetectors

**Fig. 16:**
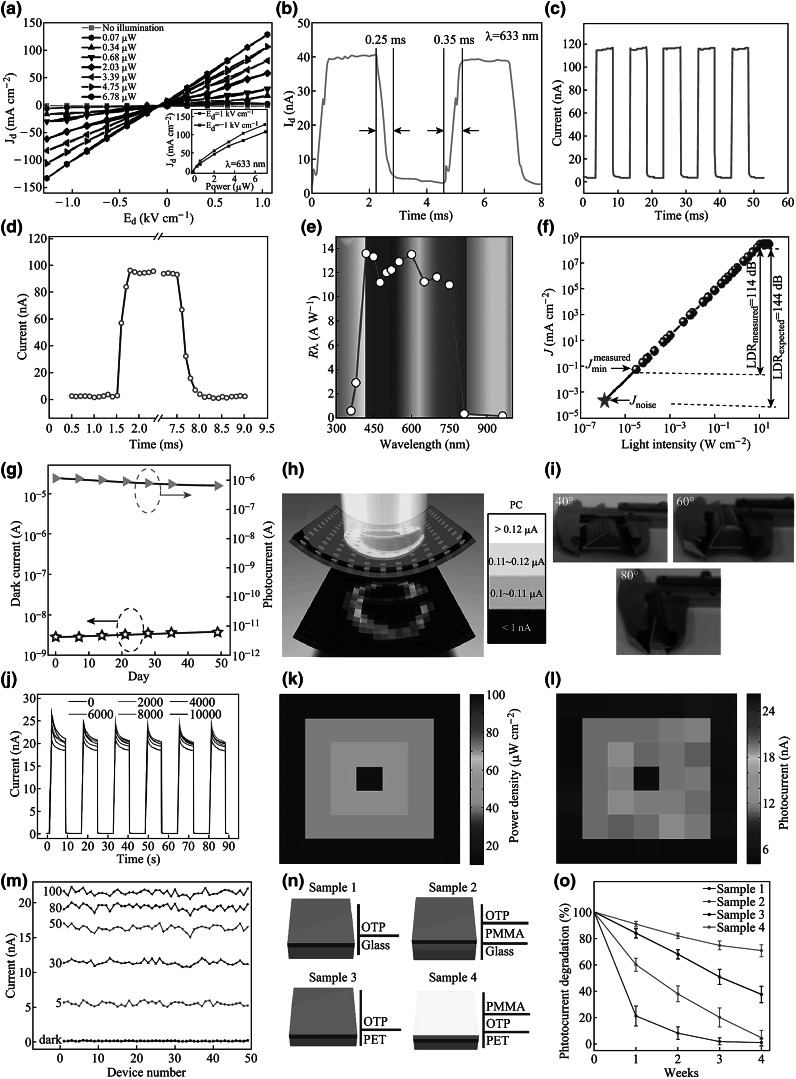
Perovskite NW photodetectors: slip coating-based NWs PDs. **a** Dark and laser-illuminated *I*–*V* curves. **b** Time-resolved photoresponse. EISA method-based device performances. **c** *I*–*t* curves. **d** High-resolution scan to one cycle of *I*–*t* curves. Doctor blading-based NW PDs. **e** Wavelength-dependent photoresponsivity of the CH_3_NH_3_PbI_3_ MW array-based photodetectors. **f** Photocurrent versus light intensity curves. **g** Variation of dark current/photocurrent with day of photodetector based on OTP MW arrays. **h** Perovskite MW PD arrays for light source mapping. NW web-based PDs for imaging. **i–j** Flexibility performances. **k**–**m** NW network PD arrays for imaging. **n**, **o** Encapsulation could help to improve the device stability. Adapted image reproduced with permission of Ref. [49, 52, 60]

PDs have a wide range of commercial and scientific applications in imaging, optical fiber communications, spectroscopy, and biomedical applications [108–110]. The commercially available PDs are commonly made by SiC, Si, and HgCdTe for UV, visible, and infrared detection, respectively [110–114]. Endre Horvá et al. first constructed two-probe detectors based on a few perovskite NWs for 633-nm light detection [49]. Under laser illumination, the light-generated electron–hole pairs caused an increase in the conductance of the material up to a factor of 300 (Fig. 16a, b). The calculated responsivity was estimated to be 5 mA W^−1^, comparable (10 times higher) to the value that has been achieved with the first prototypes of those 2D materials [115, 116]. The response time showed that rise and decay times for the on–off current are less than 500 μs, ∼10^4^ faster than the state-of-the-art PDs made of 2D TMD materials [117, 118]. Considering the nonuniformity of random NW PDs, our group utilized aligned NWs arrays to construct conductive PDs and systematically investigated the PD performances [52]. Monolayer NW-based PDs exhibited a response time of ∼0.3 ms, a responsivity of 1.3 A W^−1^, and a detectivity of 2.5 × 10^12^ Jones, which are superior to recently reported PD performances based on perovskite thin films and inorganic NWs (Fig. 16c, d).

The above studies were all based on individual PDs. However, the practical applications of PDs (for example, in imaging) require large number of pixels to construct target images. Therefore, our group first utilized perovskite PD arrays and utilized them to preliminarily implement imaging systems [58] (Fig. 16i–o). We developed a modified one-step method to implement the new NW morphology of NW webs. The PD arrays were applied in imaging and successfully obtained clear mapping of the light source signal. The network PD arrays showed superior flexibility, which could withstand wide angle (20°–80°) bending over a large number (~10,000) of cycles. Moreover, the sandwich structure (PET/OTP/PMMA) could greatly improve the device stability and the network PD arrays easily stayed intact for one month of storage in air. Our PD responsivity was further optimized by Jie’s group [60] (Fig. 16e–h). Large-scale PD arrays were fabricated by doctor blading, and the corresponding responsivity was improved to 13.5 A W^−1^. The representative results of perovskite PDs are summarized in Table 2.

**Table 2:** Overview of selected representative results of perovskite PDs [49, 52, 54–57, 119]

Materials	Configuration	Responsivity (A W^−1^)	Detectivity (Jones)	Response time	Report year
CH_3_NH_3_PbI_3_/TiO_2_ film	Photodetector	0.49 × 10^−6^	–	0.02 s	2014
CH_3_NH_3_PbI_3_ film	Photodetector	3.49	–	<0.2 s	2014
CH_3_NH_3_PbI_3−*x*_CI_*x*_ film	Photodetector	5 × 10^−3^	10^14^	160 ns	2014
CH_3_NH_3_PbI_3_ nanowires	Phototransistor	14.5	–	<500 µs	2015
CH_3_NH_3_PbI_3_ film	Photodetector	180	–	0.2 µs	2015
Graphene-CH_3_NH_3_PbI_3_ composites	Phototransistor	242	10^9^	87 ms	2015
CH_3_NH_3_PbI_3_ film	Photodetector	–	–	5.7 ± 1.0 µs	2015
CH_3_NH_3_PbI_3_ film	Photodiode	–	3 × 10^12^	<5 µs	2015
CH_3_NH_3_PbI_3_ film	Photodetector	–	7.4 × 10^12^	120 ns	2015
CH_3_NH_3_PbI_3_ film	Optocoupler	1.0	–	20 µs	2015
CH_3_NH_3_PbI_3_ nanowires	Photodetector	1.3	2.5 × 10^12^	0.3 ms	2015

### Lasing

**Fig. 17:**
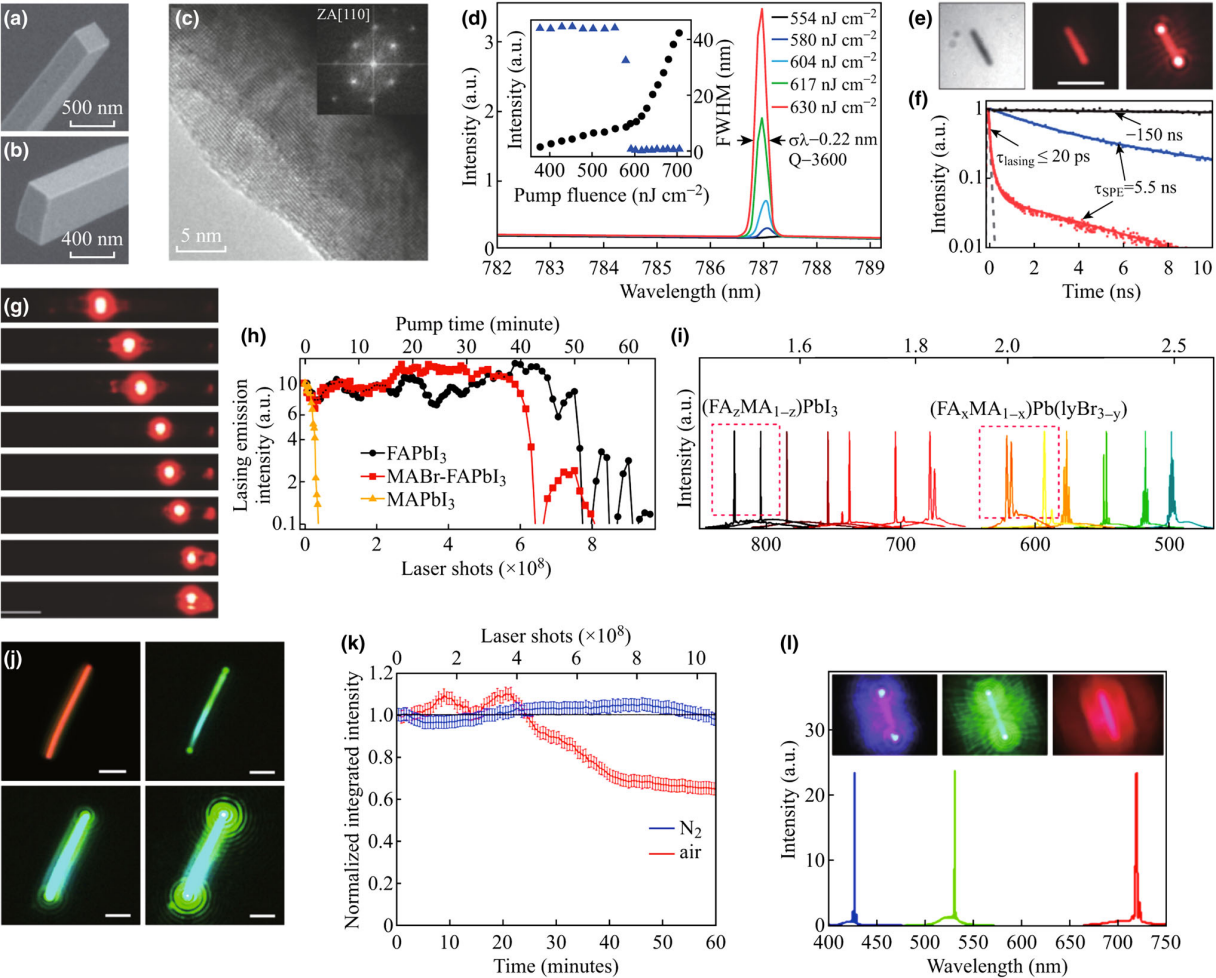
Perovskite NWs lasers. **a–f** SEM image and nanolaser performances from MAPbX_3_ NWs. **g** A set of dark-field images of a IOP MW waveguide. *Scale bar* is 10 µm. **h, i** FAPbX_3_ NW laser performances. **j–l** Full-inorganic halide perovskite NW lasers. Adapted image reproduced with permission of Ref. [67, 119, 128, 129]

Semiconductor NW lasers, owing to their ultracompact physical sizes, highly localized coherent output, and efficient waveguiding, are promising building blocks for nanoscale photonic and optoelectronic devices [120]. Each NW can serve as a waveguide along the axial direction and the two end facets form Fabry–Perot cavities for optical amplification. One of the main obstacles limiting the potential applications of semiconductor NW lasers is the high-threshold carrier density required for lasing. The amazing solar cell performance is ascribed to the long carrier lifetimes and diffusion lengths (µm range) [121–124]. These properties combined with high fluorescence yield and wavelength tunability [96, 124, 125] render lead halide perovskites as ideal materials for lasing. Song Jin et al. teamed up with X–Y. Zhu to develop a surface-initiated solution growth strategy to synthesize high-quality single-crystal NWs. NWs below the lasing threshold (*P*_Th_) showed uniform intensity from the whole NWs, and those above *P*_Th_ demonstrated strong emission with spatial interference at the two end facets [126]. They exhibited a quality factor 3600, which was more than an order higher than that of the state-of-the-art GaAs–AlGaAs core–shell NW laser operating at a temperature of 4 K [127]. In addition to single-mode lasing, they also observed multiple lasing modes from perovskite NWs. For comparison, the QYs from OIP polycrystalline thin films were <10% for spontaneous emission (SPE) and ∼15% for amplified SPE [125]. For another comparison, the state-of-the-art GaAs–AlGaAs core–shell NWs laser has a carrier lifetime of ∼440 ps and a QY of ∼0.4% [127]. These comparisons suggest that the unique lasing performance may be ascribed to the exceptionally low trap density in their single-crystal NWs. A major advantage of lead halide perovskites for lasing application was the broad wavelength tunability based on composition stoichiometry [125]. They further demonstrated the color tunability from near-infrared to blue in single-crystal perovskite NWs. By simply mixing different amounts of methylammonium iodide and bromide or bromide and chloride in the precursor solution, they successfully synthesized single-crystal NWs of MAPbBr_*y*_I_3−*y*_ and MAPbCl_*x*_Br_3−*x*_ alloys with various stoichiometries (Fig. 17a–f). Room-temperature-tunable NW lasers were also implemented by Xiong’s group, who adopted organic–inorganic perovskite NWs as the Fabry–Perot cavities to emit lasers [30].

Considering the photo- and thermal stabilities of MAPbX_3_ NW lasers, Song Jin et al. reported a low-temperature solution growth of single-crystal NWs of formamidinium lead halide perovskites (FAPbX_3_) that feature redshifted emission and better thermal stability compared to MAPbX_3_ [129]. They demonstrated optically pumped room-temperature near-infrared (∼820 nm) and green lasing (∼560 nm) from FAPbI_3_ and FAPbBr_3_ NWs with low lasing thresholds of several microjoules per square centimeter and high quality factors of approximately 1500–2300. More remarkably, the FAPbI_3_ and MABr-stabilized FAPbI_3_ NWs displayed durable room-temperature lasing under ∼10^8^ shots of sustained illumination, greatly exceeding the stability of MAPbI_3_ (∼10^7^ laser shots). They also demonstrated tunable NWs lasers in a wider wavelength region from FA-based lead halide perovskite alloys through cation and anion exchange (Fig. 17h–i).

Since organic–inorganic lead halide perovskite materials were still limited by their instability. Full-inorganic halide perovskites, cesium lead halides, offered a robust candidate without sacrificing emission tunability or ease of synthesis. Thus, Yang et al. reported the low-temperature, solution-phase growth of cesium lead halide NWs showing low-threshold lasing and high stability [119]. Upon optical excitation, Fabry–Perot lasing occurred in CsPbBr_3_ NWs with an onset of 5 μJ cm^−2^ with the NW cavity displaying a maximum quality factor of 1009 ± 5. Lasing under constant, pulsed excitation could be maintained for over 1 h, the equivalent of 10^9^ excitation cycles. Wavelength tunability in the green and blue regions and excellent stability promised those NW lasers as attractive for device applications (Fig. 17j–l). The light–matter interactions of NW lasers were investigated by Jae Kyu Song et al. [66]. The reduced mode volume of the NWs suggested strong light–matter interactions with an exciton–polariton model. The representative results of perovskite nanolasers are summarized in Table 3.

**Table 3:** Representative results of the ASE/lasing from perovskite gain media [76, 119, 123–126, 130–132]

Materials (*X* = CI^−^,Br^−^,or I^−^)	Morphology	Pump source	S.E wavelengths (nm)	Modal gain coefficient	Threshold (ASE or lasing)	Cavity type	Publication date
CH_3_NH_3_PbX_3_	Thin film	600 nm, 150 fs	500–790	40	12 (ASE)	N.A	03-2014
CH_3_NH_3_PbI_3_	Thin film	530 nm, 4 ns	780	–	10 (ASE)	N.A	08-2015
CH_3_NH_3_PbI_3−*x*_Cl_*x*_	Thin film	532 nm, 400 ps	760	–	120 (ASE) 0.2 µJ per pulse (lasing)	N.A Vertical microcavity	04-2014
CH_3_NH_3_PbI_3_	Thin film	355 nm, 2 ns	775	125	65 (ASE) 75 (lasing)	N.A Spherical WGM	10-2014
CH_3_NH_3_PbI_3_	Thin film	532,1 ns	780	–	0.32 (lasing)	DFB	12-2015
CH_3_NH_3_PbBr_3_	Square microdisk	400 nm, 120 fs	550	–	4 (lasing)	Planar WGM	04-2015
CH_3_NH_3_PbI_3−*x*_Cl_*x*_	Microplatelet	400 nm, 50 fs	760	–	40 (lasing)	Planar WGM	08-2014
CH_3_NH_3_PbI_3_	Microcrystal networks	355 nm, 0.8 ns	765	–	200 (lasing)	Random lasing	10-2014
CH_3_NH_3_PbX_3_	NWs	402 nm, 150 fs	500–780	–	0.2 (lasing)	Fabry–Perot cavity	03-2015
CsPbX_3_	NPs	400 nm, 100 fs	470–620	98	22 (ASE) 11 × 10^3^ (lasing)	N.A ring WGM	10-2015
CsPbX_3_	NPs	400 nm, 100 fs	470–640	450	5–22 (ASE) N.A (lasing)	N.A Spherical WGM Random lasing	07-2015
CsPbBr_3_	NPs	800 nm, 35 fs	520	–	12 × 10^3^ (ASE)	N.A	12-2015
CsPbBr_3_	NWs	400 nm, 150 fs	530	–	10 (lasing)	Fabry–Perot cavity	02-2016

## Summary and Outlook

In summary, we have reviewed recent developments of structure and growth of low-dimensional halide perovskites and their applications for high-performance optoelectronic devices. Their remarkable intrinsic properties and high degree of structural flexibility allow the potential beyond academic curiosity and industrial interest. In particular, the high QY, narrow emission, high exciton binding energies, color tunability, and facile solution processing at low temperatures have been outlined. Beyond applications as active layers for emitters and optical gain materials, low-dimensional halide perovskites may also be incorporated in currently existing technologies as a candidate to replace phosphors. As the field grows from its infancy stage, various kinds of heterojunctions, architectures, and processing conditions are probable to promote rapid development. For the routes toward commercialization, the key issues such as the optoelectronic properties (e.g., excitation dynamics, nonradiative recombination), working principles, and degradation mechanisms need better understanding. Owing to the successes in photovoltaics, it is reasonable to expect that the research in other perovskite optoelectronics will intensify and usher in a bright future for PDs, lasers, and light-emitting devices (Table 4).

**Table 4:** Stability improvement strategies for perovskite [103, 130–136]

Perovskite	Passivation materials	Method	Report time
CsPbX_3_	The incorporation of poly(maleic anhydride-alt-l-octadecene) (PMA)	Tightening the ligand binding	2016
CsPbX_3_	A polyhedral oligomeric silsesquioxane (POSS)	Surface protection	2016
CsPbX_3_	(3-aminopropyl)triethoxysilane (APTES)	A silica matrix	2016
CsPbX_3_	–	X-ray illumination	2016
CsPbX_3_	–	Intermolecular C = C bonding	2016
CH_3_NH_3_PbBr_3_	SiO_2_	Coupling with perovskites	2016
FAPbBr3	Hydrophobic association	Capping nanocrystals	2016
CsPbX_3_	Chloride	Chloride doping	2016

For low-dimensional halide perovskite synthesis, the perovskite formation processes play a paramount role in determining their final device performances. They can be prepared by a variety of techniques via the competition between in situ transformation and dissolution–crystallization mechanisms. Moreover, the use of capping ligands and solvent engineering can help to tailor the shape of perovskite crystals. Emerging applications of these randomly distributed perovskite units require the achievement of controllable alignment in order to match the planar microelectronic fabrication techniques.

Beyond PV applications, low-dimensional perovskite materials with high crystallinity, emission efficiency, and benign defects enable the fabrication of LEDs, lasers, PDs, and other optoelectronic/microelectronic devices. Furthermore, their high atomic number and high density may find applicability to high-energy radiation detection. Additionally, perovskites exhibit interesting ferroelectric properties, potentially due to the free rotation of the polar organic species, which further bolsters their potential for switchable electronics and memory devices. Despite the novel properties of low-dimensional perovskites, several crucial challenges, including toxicity and instability, limit wide industrial application. Limited success has been reported for the replacement of Pb with environmental friendly elements. Nontoxic Sn shares relatively similar properties to Pb in hybrid perovskites and is theoretically expected to yield more efficient performances. However, Sn-based perovskite solar cells experience more serious instability due to the naturally favorable oxidation of Sn(II), which inevitably requires advanced encapsulation techniques. Apart from the toxicity, Pb-based perovskites experience inherent instability under long-term operation, which is even more urgent to address. The degradation mechanisms of perovskites upon exposure to thermal, moisture, UV, and mechanical conditions require a reliable strategy in order to improve material stability. Encouraging results have been demonstrated through interface modifications between transport materials and electrodes [121]. Optimized devices have realized stable working times over 1000 h. However, considering the durability requirements of 20 years operation for solar cells, there is still much work required to improve the intrinsic properties of halide perovskites and more stable device structures. From an academic research perspective, there have been great success for the synthesis and applications of halide perovskites. Further research efforts are expected to focus on: (1) deep understanding of the structure–property relationships of the entire hybrid material system to guide the rational design and careful manipulation of the optoelectronic properties and (2) introducing new ideas to break the trade-off between the halide perovskite novelties and the working stability. Under the intensely driven research, the future development of environmentally friendly and reliably working perovskite devices is hopeful.
